# Supplementary material for: Early Results of a Screening Program for Skin Cancer in Liver Transplant Recipients: A Cohort Study
Source: Cancers (Basel). 2024 Mar 20;16(6):1224. doi: 10.3390/cancers16061224 (PMC10969135; doi:10.3390/cancers16061224)
Supplement: Supplementary file 1 [file cancers-16-01224-s001.zip › cancers-2867977-supplementary.pdf]

Table S1. Characteristics of liver transplant recipients with skin cancer/preneoplastic lesions

**Nr.**	**Sex**	**Birthyear**	**Age at LTX**	**Skin cancers & preneoplastic lesions during screening period**, year (n)	**Previous skin cancer / preneoplastic lesions**	**Immunosuppressive treatment**	**LTX indication**	**Smoking status**
**1**	Male	1939	58	Actinic keratosis: 2018 (1), 2021 (2)<br>SCC: 2021 (2)	Actinic keratosis: 2011 (1), 2017 (2)<br>BCC: 2013 (1)	Cyclosporin, Azathioprine, Prednisolone	Primary sclerosing cholangitis	Former
**2**	Male	1959	53	Actinic keratosis: 2020 (1)<br>BCC: 2019 (1)	Actinic keratosis: 2017 (1)	Tacrolimus, Mycophenolate	Cirrhosis (alcoholic / cryptogenic	Former
**3**	Female	1952	63	Actinic keratosis: 2019 (3) 2020 (3), 2021 (1)<br>Bowen's Disease: 2020 (1)<br>SCC: 2021 (1)	-	Tacrolimus, Mycophenolate, Prednisolone	Primary sclerosing cholangitis	Current
**4**	Male	1966	45	Actinic keratosis: 2018 (1), 2020 (1), 2021 (1)	Bowen's Disease: 2012 (1)	Cyclosporin, Mycophenolate, Prednisolone	Primary sclerosing cholangitis	Never
**5**	Male	1951	44	BCC: 2018 (1)	BCC: 2017 (1)	Cyclosporin	Cirrhosis (alcoholic / cryptogenic	Former
**6**	Female	1975	40	Bowen's Disease: 2019 (1)	BCC: 2017 (1)	Tacrolimus, Prednisolone	Primary sclerosing cholangitis	Never
**7**	Female	1983	26	BCC: 2018 (1)	-	Tacrolimus, Azathioprine, Prednisolone	Autoimmune hepatitis	Never
**8**	Female	1946	30	BCC: 2018 (1), 2019 (1), 2020 (1)	BCC: 2001 (3), 2002 (1), 2010 (1), 2014 (2), 2017 (2)<br>Actinic keratosis: 2010 (1), 2011 (1), 2012 (1)	Everolimus, Prednisolone	Polycystic liver disease	Never
**9**	Female	1960	46	Bowen's Disease: 2019 (1)	-	Tacrolimus, Prednisolone	Cirrhosis (alcoholic / cryptogenic	Unknown
**10**	Male	1943	51	Actinic keratosis: 2018 (3), 2021 (3)<br>Bowen's Disease: 2018 (1)<br>BCC: 2020 (1)<br>SCC: 2018 (2)	-	Tacrolimus, Mycophenolate, Prednisolone	Cirrhosis (alcoholic / cryptogenic	Never
**11**	Male	1946	54	BCC: 2019 (2), 2020 (1) 2021 (1)	Actinic keratosis: 2017 (1)	Tacrolimus, Mycophenolate	Primary sclerosing cholangitis	Unknown
**12**	Male	1947	66	SCC: 2018 (1)	Actinic keratosis: 2017 (1)	Tacrolimus, Mycophenolate	Primary biliary cholangitis	Former
**13**	Female	1950	42	Actinic keratosis: 2021 (1)<br>BCC: 2019 (2), 2020 (3), 2021 (2)	BCC: 1994 (1), 1995 (1), 1997 (1), 1998 (2), 1999 (3), 2000 (2), 2009 (1), 2011 (1), 2012 (1), 2016 (3), 2017 (2)<br>Actinic keratosis: 1997 (2), 1998 (1), 2010 (1), 2014 (2), 2015 (1), 2016 (1)<br>Bowen's Disease: 1998 (1), 2016 (1)	Mycophenolate, Prednisolone	Polycystic liver disease	Never

					SCC: 2004 (1), 2012 (1), 2016 (2)			
**14**	Male	1953	42	Actinic keratosis: 2019 (1)	Actinic keratosis: 2005 (1)<br>SCC: 2005 (2), 2006 (1)<br>Bowen's Disease: 2006 (1)	Cyclosporin, Azathioprine	Hepatitis B virus	Former
**15**	Female	1971	20	Actinic keratosis: 2019 (1)<br>BCC: 2019 (2)<br>SCC: 2019 (1)	BCC: 2006 (1)<br>SCC: 2017 (1)	Tacrolimus, Mycophenolate	Epitheloid Hemangio-endothelioma	Current
**16**	Female	1942	50	Actinic keratosis: 2019 (1)	-	Tacrolimus, Azathioprine, Prednisolone	Primary biliary cholangitis	Former
**17**	Male	1955	53	Actinic keratosis: 2019 (1)<br>BCC: 2020 (1)	BCC: 2013 (1)<br>MM: 2017 (1)	Tacrolimus, Mycophenolate	Autoimmune hepatitis	Never
**18**	Male	1960	47	Actinic keratosis: 2020 (2)<br>SCC: 2019 (1)	BCC: 2014 (1)	Tacrolimus, Mycophenolate	Cirrhosis (alcoholic / cryptogenic	Never
**19**	Male	1946	57	Actinic keratosis: 2019 (1), 2020 (2), 2021 (2)<br>SCC: 2020 (1), 2021 (2)	Actinic keratosis: 2013 (2), 2014 (1)<br>SCC: 2014 (1)	Everolimus, Mycophenolate	Cirrhosis (alcoholic / cryptogenic<br>& HCC	Former
**20**	Male	1965	41	BCC: 2021 (2)	BCC: 2012 (1), 2015 (2)	Tacrolimus, Mycophenolate, Prednisolone	Primary sclerosing cholangitis	Never
**21**	Male	1961	46	BCC: 2018 (1)	-	Tacrolimus, Mycophenolate, Prednisolone	Primary sclerosing cholangitis	Never
**22**	Male	1975	32	BCC: 2020 (1)	-	Tacrolimus, Mycophenolate, Prednisolone	Primary sclerosing cholangitis	Never
**23**	Male	1961	47	Bowen's Disease: 2019 (1), 2020 (1), 2021 (1)<br>BCC: 2021 (3)	-	Tacrolimus, Mycophenolate	Iatrogenic -antabus induced hepatitis	Current
**24**	Male	1958	49	Actinic keratosis: 2021 (2)	-	Tacrolimus, Mycophenolate	Cirrhosis (alcoholic / cryptogenic<br>& HCC	Never
**25**	Female	1949	60	Actinic keratosis: 2020 (2), 2021 (1)<br>SCC: 2021 (1)	BCC: 2012 (2), 2015 (1)	Tacrolimus, Mycophenolate, Prednisolone	Primary biliary cholangitis	Current
**26**	Male	1955	54	BCC: 2018 (2)	SCC: 2011 (3), 2015 (1), 2016 (1)	Tacrolimus, Mycophenolate, Prednisolone	Primary sclerosing cholangitis	Never
**27**	Male	1966	45	BCC: 2021 (1)	-	Tacrolimus, Mycophenolate	Primary sclerosing cholangitis	Never
**28**	Male	1953	63	BCC: 2020 (2)	-	Tacrolimus, Mycophenolate	HCC	Current
**29**	Female	1953	64	BCC: 2019 (1), 2020 (1)<br>SCC: 2021 (1)	-	Tacrolimus, Mycophenolate	HCC	Never
**30**	Female	1948	51	Actinic keratosis: 2018 (1), 2020 (1)	-	Cyclosporin, Mycophenolate	Cirrhosis (alcoholic / cryptogenic)	Current

							& metabolic liver disease	
**31**	Male	1939	62	Actinic keratosis: 2020 (2), 2021 (1)<br>BCC: 2021 (1)<br>SCC: 2019 (3)	SCC: 2005 (1), 2007 (2), 2008 (1) BCC: 2005 (1), 2006 (2) Actinic keratosis: 2006 (2), 2007 (2), 2008 (2), 2010 (1), 2012 (1), 2015 (2), 2016 (1)	Cyclosporin, Mycophenolate	Cirrhosis (alcoholic / cryptogenic)	Current
**32**	Female	1957	39	Actinic keratosis: 2020 (1)	-	Tacrolimus, Azathioprine, Prednisolone	Autoimmune hepatitis	Never
